# Medication Availability for Alcohol Use Disorder in Substance Use Disorder Treatment Facilities

**DOI:** 10.1001/jamanetworkopen.2025.51563

**Published:** 2026-01-12

**Authors:** Yuji Mizushima, Jonathan Cantor, Ryan K. McBain, Fang Zhang, Alyssa Burnett, Ensheng Dong, Aaron Kofner, Joshua Breslau, Bradley D. Stein, Hao Yu

**Affiliations:** 1 Economics, Sociology, and Statistics Department, RAND, Santa Monica, California; 2 Behavioral and Policy Sciences Department, RAND, Arlington, Virginia; 3 Brigham & Women’s Hospital, Boston, Massachusetts; 4 Harvard Pilgrim Health Care Institute, Boston, Massachusetts; 5 Department of Population Medicine, Harvard Medical School, Boston, Massachusetts; 6 Engineering and Applied Sciences Department, RAND, Arlington, Virginia; 7 Data Science and Technology Department, RAND, Arlington, Virginia; 8 Behavioral and Policy Sciences Department, RAND, Pittsburgh, Pennsylvania

## Abstract

**Importance:**

Alcohol use disorder (AUD) is a major public health concern; medications for AUD (MAUD) are an effective form of treatment but remain underused. Identifying MAUD access trends and the characteristics of counties with limited availability can inform targeted efforts to expand treatment capacity.

**Objective:**

To examine trends in geographic availability of MAUD at US substance use disorder treatment facilities (SUDTFs) from 2017 to 2023 and assess county characteristics associated with SUDTFs offering MAUD.

**Design, Setting, and Participants:**

This nationwide cross-sectional study used data from the Mental Health and Addiction Treatment Tracking Repository, which includes longitudinal data on licensed SUDTFs and whether they offer MAUD (acamprosate, disulfiram, or naltrexone), to quantify trends in MAUD availability at SUDTFs from January 2017 to December 2023.

**Main Outcomes and Measures:**

The primary outcome was a county-year indicator for whether at least 1 SUDTF in the county offered MAUD. Explanatory county variables included rurality, percentage of traffic fatalities involving alcohol, percentage of the population that drank excessively, percentage of uninsured individuals, poverty rate, percentage of individuals over age 65 years, and percentage of non-Hispanic White individuals. Univariate logistic regressions with state and year fixed effects were used to explore associations between county characteristics and the probability that a county had any SUDTFs offering MAUD.

**Results:**

Across 22 000 county-years in a total of 3153 counties, the mean (SD) percentage of counties with at least 1 SUDTF offering MAUD increased from 34.12% (47.42%) in 2017 to 43.88% (49.63%) in 2021, but growth plateaued after 2021. Lower MAUD presence in a county was associated with rural-adjacent (difference, −22.40 percentage points [pp]; 95% CI, −24.43 to −20.38 pp) and rural-remote (−23.64 pp; 95% CI, −25.72 to −21.56 pp) relative to metropolitan county status as well as with a higher poverty rate (−0.66 pp; 95% CI, −0.93 to −0.38 pp), greater percentage of individuals aged 65 years or older (−2.33 pp; 95% CI, −3.02 to −1.65 pp), and higher proportion of non-Hispanic White individuals (−0.58 pp; 95% CI, −0.71 to −0.46 pp), whereas greater prevalence of binge drinking (difference, 1.90 pp; 95% CI, 1.26-2.54 pp) and a higher percentage of college-educated individuals (1.28 pp; 95% CI, 1.13-1.43) were associated with higher MAUD presence.

**Conclusions and Relevance:**

In this cross-sectional study, the proportion of SUDTFs offering MAUD increased from 2017 to 2021, but growth then plateaued. Policies supporting the expansion of MAUD-providing facilities, particularly in underserved counties, may be needed to address persistent gaps in access.

## Key Points

**Question:**

What were the trends in availability of medications for alcohol use disorder (MAUD) in US substance use disorder treatment facilities (SUDTFs) from 2017 to 2023, and which county characteristics were associated with their presence?

**Findings:**

In this cross-sectional study of SUDTFs in 3153 counties across 22 000 county-years, the proportion of counties with at least 1 SUDTF offering MAUD increased from 34% in 2017 to 44% in 2021 but did not rise further through 2023. Rural, smaller, and socioeconomically disadvantaged counties were significantly less likely to have MAUD-offering SUDTFs.

**Meaning:**

Policies supporting MAUD-providing facilities may be needed to address persistent gaps in access.

## Teaser

This cross-sectional study assesses trends in availability of medications for alcohol use disorder in US substance use disorder treatment facilities from 2017 to 2023 by demographic and socioeconomic county characteristics.

## Introduction

In 2023 alone, 28.9 million people aged 12 years or older in the US had alcohol use disorder (AUD).^1^ Heavy alcohol consumption corresponds to preventable alcohol-related harms.^2^ The number of alcohol-related deaths for individuals 16 years of age or older increased by roughly 50 percent between 1999 and 2017 (16.9 to 25.5 per 100 000 population).^3^ Between 2019 and 2020, there was a 25.5% increase in the number of deaths involving alcohol.^4^ More than 1 million individuals in the US are projected to die from alcohol-related liver disease between 2019 and 2040.^5^

Elevated rates of AUD and alcohol-related harms continue to occur despite effective medication treatment for AUD.^6^ There are 3 treatments approved by the US Food and Drug Administration (FDA) for AUD: naltrexone, acamprosate, and disulfiram.^7^ A recent systematic review and meta-analysis of 118 clinical trials concluded that oral naltrexone and acamprosate both can serve as first-line pharmacotherapies for AUD, with weaker evidence supporting the efficacy of disulfiram.^8^ Naltrexone is an opioid antagonist for alcohol dependence^9^ that can be administered orally daily or as a single-dose injectable lasting 30 days.^10^ In contrast, acamprosate and disulfiram are only administered orally daily. Federal and state policies, including the Mental Health Parity and Addiction Equity Act and the Affordable Care Act,^11^ have attempted to improve access to medications for alcohol use disorder (MAUD).^12,13,14^ Yet, rates of MAUD use in the US remain very low—only 1.9 percent among individuals with AUD in 2023.^1^

Specialty substance use disorder treatment facilities (SUDTFs) represent an important setting for AUD treatment, providing a structured and supportive environment for patients to receive care.^15^ These facilities often care for patients who have comorbid behavioral health conditions^16^ and can offer effective forms of integrated care.^17^ This makes specialty facilities a critical entry point for treating AUD and potentially using MAUD. In 2021, SUDTFs in the US received 439 755 treatment admissions with alcohol listed as the primary substance—roughly one-third of all SUDTF admissions.^16^

Currently, there is limited national longitudinal research on MAUD availability at SUDTFs. Previous research has found that inpatient and hospital-based programs are more likely to offer MAUD compared with outpatient or residential programs.^18^ One recent study using the 2022 National Substance Use and Mental Health Services Survey found that 45% of specialty facilities offered 1 form of MAUD, and 22% offered all 3 forms.^13^ However, the study used only 1 year of data and did not examine county-level factors associated with offering MAUD. Another study used data from the National Directory of Drug and Alcohol Abuse Treatment Facilities from 2016 to 2019 and found that facilities offering MAUD were more likely to be available in urban counties but not in counties with a higher prevalence of excessive drinking.^19^

The present study calculated trends in the availability of MAUD nationwide from 2017 to 2023, identifying county-level characteristics associated with MAUD availability. This inquiry provides important information on the AUD treatment landscape, including which facility characteristics and regions may require further incentives for expanding MAUD.

## Methods

### Measures

In this cross-sectional study, we used data from the Mental Health and Addiction Treatment Tracking Repository (MATTR) to quantify trends in the availability of MAUD at SUDTFs from January 2017 to December 2023. MATTR contains annualized composite information from the National Directory of Drug and Alcohol Abuse Treatment Facilities, released by the Substance Abuse and Mental Services Administration.^20^ The facility listings are geocoded and linked across years. The data are based on responses to the National Survey of Substance Abuse Treatment Services or the National Substance Use and Mental Health Services Survey. Each facility’s geocoded location was linked to its county’s Federal Information Processing Standards code. The Harvard Pilgrim Health Care Institute’s institutional review board considered this cross-sectional study not human participants research; thus, it did not require informed consent. This study followed the Strengthening the Reporting of Observational Studies in Epidemiology (STROBE) reporting guideline.

MATTR contains measures of the types of care offered by SUDTFs, including types of MAUD. Specifically, since 2017, facilities have been asked if they offer acamprosate, disulfiram, and/or naltrexone. Our main measure for MAUD availability was a county-year–level indicator variable equal to 1 if a county had at least 1 SUDTF offering any of these 3 MAUD and 0 otherwise.

County-level covariates were chosen a priori based on plausible demographic and socioeconomic characteristics that are likely associated with facilities offering MAUD. To measure demographic characteristics, we included the percentage of the population aged 65 years or older, the percentage that was non-Hispanic White, the total population, and county rurality. We included the proportion aged 65 years or older to test the association between broader structural demographic aging of a county and the availability of MAUD at SUDTFs—an important factor given prior studies pointing to increasing AUD prevalence, greater sensitivity to its adverse health effects, and underdiagnosis among older adults.^21,22,23^ For socioeconomic factors, we included the percentage of the population in a county living below the federal poverty level, the percentage uninsured, and the percentage with an educational level of bachelor’s degree or higher. Race and ethnicity were self-reported by respondents in the American Community Survey (ACS) 5-year estimates. Categories included Hispanic; non-Hispanic Black (hereafter, *Black*); non-Hispanic White (hereafter, *White*); and other (included non-Hispanic American Indian or Alaska Native; non-Hispanic Asian; non-Hispanic Native Hawaiian or Other Pacific Islander; multiracial; and some other race, non-Hispanic). All other covariates were constructed using data from the ACS 5-year estimates (2019-2023).^24^

Using 2023 Rural-Urban Continuum Codes (RUCC),^25^ counties were designated as metropolitan (codes 1, 2, or 3), rural adjacent (codes 4, 6, or 8), or rural remote (codes 5, 7, or 9). To account for Connecticut’s county restructuring in 2022, we used the 2023 RUCC codes for 2022-2023 and the 2013 RUCC codes for earlier years (2017-2021) for Connecticut.^26^ We also included 2 markers of problematic alcohol use: the annual percentage of driving deaths involving alcohol impairment was obtained from the Fatality Analysis Reporting System,^27^ and the annual percentage of adults reporting binge or heavy drinking (age-adjusted) was obtained from the Behavioral Risk Factor Surveillance System.^28^ All covariates varied at the county-year level except rurality indicators, which were time-invariant.

### Statistical Analysis

First, we calculated the percentage of counties with any SUDTFs offering MAUD in 2017 through 2023. Second, we examined whether MAUD geographic availability evolved by calculating the percentage of counties with any SUDTFs offering MAUD and the number of SUDTFs offering MAUD in a county for each year from 2017 to 2023. We computed unadjusted means of county characteristics by whether a county had any SUDTFs that offered MAUD. We calculated the difference in means across county MAUD status separately for each county-level measure. A *t* test for each difference was conducted to assess statistical significance.

Third, we performed separate univariate logistic regressions that assessed the associations between county characteristics and the presence of any MAUD-offering SUDTFs within the county. The models adjusted for state and year fixed effects, implying comparisons across counties within 357 state-year pairs. We clustered SEs at the state level and report marginal effect estimates as percentage point (pp) differences.

We also conducted several sensitivity checks. First, we dropped state fixed effects to allow for cross-county comparisons regardless of the state within which a county is located. Second, we used negative binomial regressions to assess the association between county characteristics and the number of SUDTFs offering MAUD in a county, conditional on the presence of any SUDTF offering MAUD. We used a negative binomial model rather than a Poisson model due to overdispersion in the outcome.

All analyses were completed using Stata software, version 18 (StataCorp LLC). All statistical tests were 2-sided, and *P* < .05 was considered statistically significant. To account for multiple hypothesis testing, *P* values were adjusted using the Bonferroni correction, calculated as the observed *P* value multiplied by the number of hypothesis tests, with adjusted *P* values capped at greater than .99.

## Results

### Descriptive Statistics

After excluding overseas territories, a total of 3153 unique counties or comparable units (eg, boroughs in Alaska) were analyzed. Across 22 000 US county-years from 2017 to 2023, Table 1 shows that the mean (SD) total county population was 104 020 (331 830), with a mean (SD) of 37.74% counties (48.47%) classified as metropolitan, 33.26% (47.12%) as rural adjacent, and 29.00% (45.38%) as rural remote. A mean (SD) of 28.68% (15.32%) of driving deaths involved alcohol, and 18.03% (3.39%) of adults reported binge drinking, 9.83% (5.14%) were uninsured, 14.90% (6.27%) lived below the poverty level, 22.57% (9.79%) had a bachelor’s degree or higher, and 19.04% (4.78%) were aged 65 years or older. A mean (SD) of 8.83% (14.33%) of individuals were Black, 9.60% (13.89%) were Hispanic, 75.64% (20.20%) were White, and 5.93% (8.99%) were other race and ethnicity. Among SUDTFs with MAUD, a mean (SD) of 61.65% (48.63%) offered acamprosate, 58.25% (49.32%) offered disulfiram, and 91.27% (28.23%) offered naltrexone. There were 232 missing observations in the percentage of driving deaths involving alcohol impairment (1.05% of 22 000 observations) and 34 missing observations in binge drinking (0.15% of 22 000 observations). Missingness in the percentage of driving deaths due to alcohol impairment was less common in counties with SUDTFs offering MAUD (6 of 8799 observations [0.07%]) compared with counties without facilities offering MAUD (226 of 13 201 observations [1.71%]). Missingness in binge drinking observations was less common in counties with SUDTFs offering MAUD (0 of 8799 observations) compared with counties without facilities offering MAUD (34 of 13 201 observations [0.26%]).

**Table 1.** Characteristics of Counties With vs Without Any SUDTFs Offering MAUD, 2017-2023

Characteristic^b^	Counties^a^						Mean difference (95% CI)^c^	*P* value^d^	Bonferroni-adjusted *P* value^d^
	All		With facilities offering MAUD		Without facilities offering MAUD				
	County-year observations, No.	Mean (SD)	County-year observations, No.	Mean (SD)	County-year observations, No.	Mean (SD)			
Total population, No.	22 000	104 020 (331 830)	8799	220 100 (500 230)	13 201	26 650 (41 900)	193 450 (184 870 to 202 030)	<.001	<.001
Rurality^e^									
Metropolitan	22 000	37.74 (48.47)	8799	57.08 (49.50)	13 201	24.85 (43.21)	32.23 (30.99 to 33.46)	<.001	<.001
Rural adjacent	22 000	33.26 (47.12)	8799	26.21 (43.98)	13 201	37.96 (48.53)	−11.75 (−13.01 to −10.49)	<.001	<.001
Rural remote	22 000	29.00 (45.38)	8799	16.72 (37.32)	13 201	37.19 (48.33)	−20.48 (−21.67 to −19.28)	<.001	<.001
Driving deaths involving alcohol	21 768	28.68 (15.32)	8793	28.40 (10.97)	12 975	28.87 (17.67)	−0.47 (−0.89 to −0.06)	.03	.35
Binge drinking prevalence	21 966	18.03 (3.39)	8799	18.58 (3.23)	13 167	17.65 (3.45)	0.93 (0.84 to 1.02)	<.001	<.001
Uninsured	22 000	9.83 (5.14)	8799	8.53 (4.07)	13 201	10.70 (5.57)	−2.17 (−2.30 to −2.03)	<.001	<.001
Below poverty level	22 000	14.90 (6.27)	8799	13.90 (5.29)	13 201	15.57 (6.77)	−1.67 (−1.84 to −1.50)	<.001	<.001
With bachelor’s degree or higher	22 000	22.57 (9.79)	8799	26.96 (10.92)	13 201	19.64 (7.67)	7.33 (7.08 to 7.57)	<.001	<.001
Age ≥65 y	22 000	19.04 (4.78)	8799	17.86 (4.23)	13 201	19.83 (4.97)	−1.97 (−2.10 to −1.84)	<.001	<.001
Race and ethnicity									
Hispanic	22 000	9.60 (13.89)	8799	9.66 (11.94)	13 201	9.56 (15.05)	0.10 (−0.28 to 0.47)	.61	>.99
Non-Hispanic Black	22 000	8.83 (14.33)	8799	8.51 (12.40)	13 201	9.04 (15.49)	−0.53 (−0.92 to −0.14)	.007	.10
Non-Hispanic White	22 000	75.64 (20.20)	8799	75.24 (18.80)	13 201	75.90 (21.08)	−0.66 (−1.20 to −0.11)	.02	.26
Other^f^	22 000	5.93 (8.99)	8799	6.58 (7.19)	13 201	5.50 (9.99)	1.09 (0.85 to 1.33)	<.001	<.001

Abbreviations: MAUD, medications for alcohol use disorder; SUDTF, substance use disorder treatment facility.

^a^ Data are at the county-year level.

^b^ Characteristics are presented as prevalence percentages unless otherwise indicated.

^c^ A *t* test for difference in means between counties with a facility offering MAUD and counties without a facility offering MAUD was conducted. Each difference was estimated separately for each covariate.

^d^ *P* values were multiplied by 14 and capped at .99 for Bonferroni adjustments.

^e^ Metropolitan includes counties with 2023 Rural-Urban Continuum Codes 1-3; rural adjacent, codes 4, 6, and 8; and rural remote, codes 5, 7, and 9. Connecticut used 2023 Rural-Urban Continuum Codes for 2022-2023 and 2013 codes for 2017-2021.

^f^ Includes non-Hispanic American Indian or Alaska Native; non-Hispanic Asian; non-Hispanic Native Hawaiian or Other Pacific Islander; multiracial; and some other race.

Table 1 also presents unadjusted characteristics of counties with 1 or more facilities offering MAUD and those without such facilities from 2017 to 2023. On average, counties with at least 1 MAUD-offering facility had substantially larger populations (mean [SD], 220 100 [500 230] vs 26 650 [41 900]; *P* < .001) and were more likely to be metropolitan (mean [SD], 57.08% [49.50%] vs 24.85% [43.21%]; *P* < .001). They also had a lower mean (SD) percentage of rural-adjacent counties (26.21% [43.98%] vs 37.96% [48.53%]; *P* < .001) and rural-remote counties (16.72% [37.32%] vs 37.19% [48.33%]; *P* < .001). Compared with counties without MAUD-offering facilities, those with such facilities had a lower mean (SD) percentage of driving deaths involving alcohol (28.40% [10.97%] vs 28.87% [17.67%]; *P* = .03), but this difference was not significant after Bonferroni adjustment (*P* = .35). Counties with MAUD-offering facilities had a higher mean (SD) prevalence of binge drinking (18.58% [3.23%] vs 17.65% [3.45%]; *P* < .001), a smaller share of uninsured residents (8.53% [4.07%] vs 10.70% [5.57%]; *P* < .001), lower poverty rates (13.90% [5.29%] vs 15.57% [6.77%]; *P* < .001), and a greater percentage of residents with a bachelor’s degree or higher (26.96% [10.92%] vs 19.64% [7.67%]; *P* < .001). They also had a smaller mean (SD) proportion of residents aged 65 years or older (17.86% [4.23%] vs 19.83% [4.97%]; *P* < .001). The mean (SD) percentage of non-Hispanic White residents was marginally lower in counties with MAUD-offering facilities (75.24% [18.80%] vs 75.90% [21.08%]; *P* = .02), but this association was not statistically significant after Bonferroni correction (*P* = .26). All other comparisons remained significant after adjustment.

**Figure 1.**
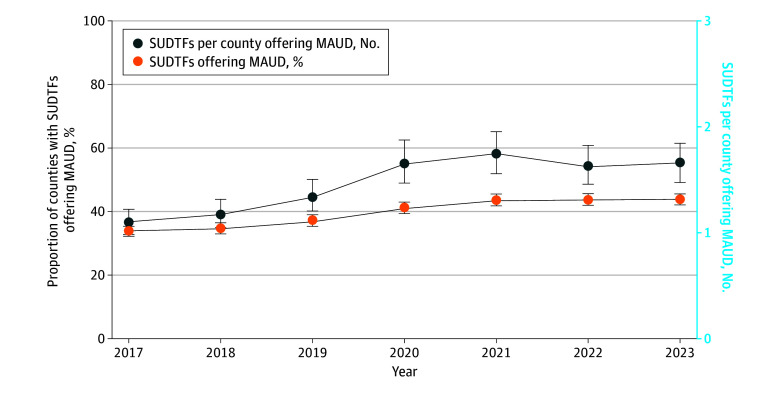
Counties With Substance Use Disorder Treatment Facilities (SUDTFs) Offering Medications for Alcohol Use Disorder (MAUD) and Number of SUDTFs Offering MAUD From 2017 to 2023 Whiskers represent 95% CIs for the mean coefficients. *t* Tests comparing the mean coefficients for 2021 vs 2022 and 2021 vs 2023 were not statistically significant for either outcome.

**Figure 2.**
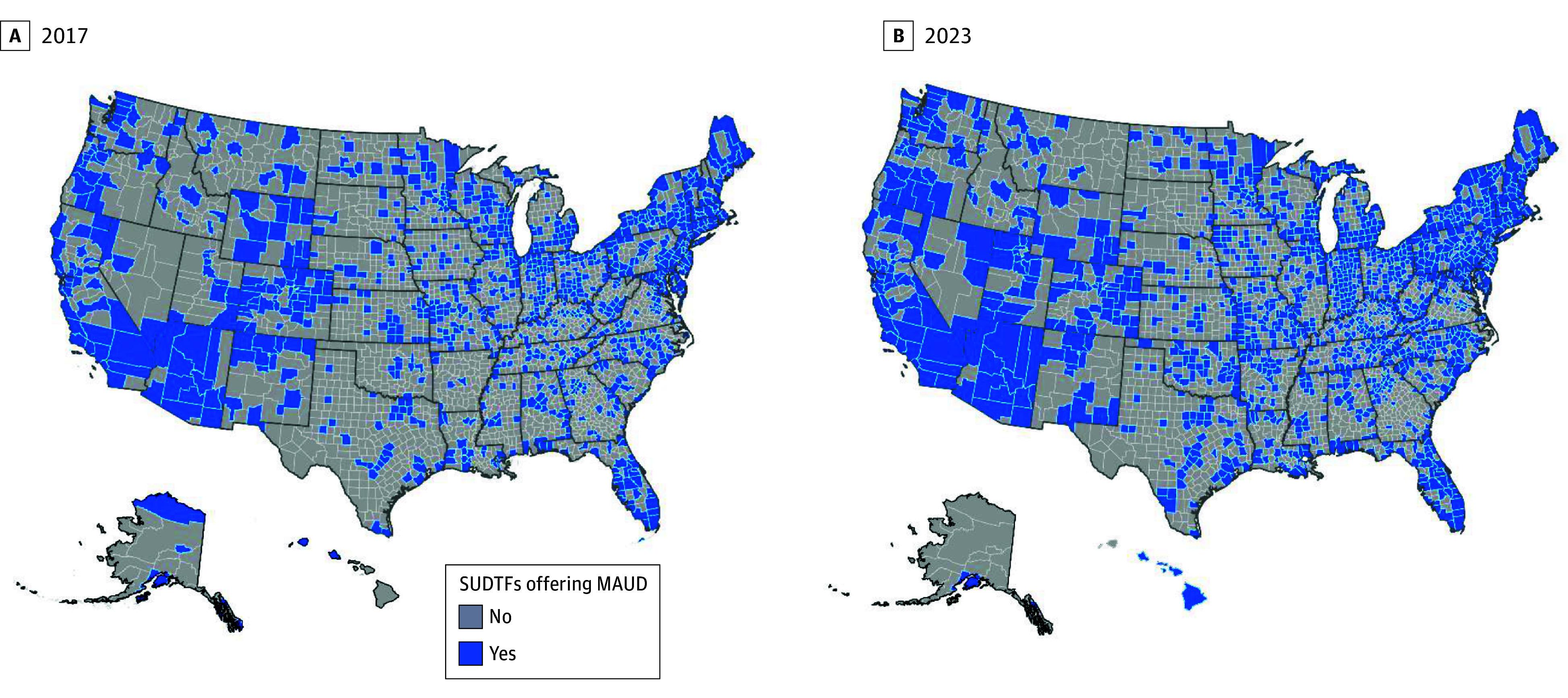
Counties With Substance Use Disorder Treatment Facilities (SUDTFs) Offering Medications for Alcohol Use Disorder (MAUD) in 2017 and 2023

Figure 1 shows that the mean (SD) percentage of counties with any SUDTF offering MAUD increased from 34.12% (47.42%) in 2017 to 43.88% (49.63%) in 2021, plateauing between 2021 and 2023. *t* Tests comparing the coefficients for 2021 vs 2022 and 2021 vs 2023 were not statistically significant. This trend was similar when evaluating the number of SUDTFs offering MAUD in a county from 2017 to 2023: *t* tests comparing the coefficients for 2021 vs 2022 and 2021 vs 2023 were not statistically significant. A similar trend was observed when examining the number of SUDTFs offering MAUD per 100 000 population, as reported in the eFigure in Supplement 1. As shown in Figure 2, a large percentage of counties with any SUDTFs offering MAUD was concentrated in coastal regions of the US. The geographic availability of facilities offering MAUD also increased between 2017 and 2023.

### Regression Analyses

In Table 2, we report results of separate univariate regressions for each county characteristic, adjusting for year and state fixed effects. Compared with metropolitan counties, rural-adjacent counties had lower availability of facilities offering MAUD (difference, −22.40 pp; 95% CI, −24.43 to −20.38 pp; *P* < .001), as did rural-remote counties (−23.64 pp; 95% CI, −25.72 to −21.56 pp; *P* < .001). A 1-pp increase in binge-drinking prevalence was associated with a higher likelihood of having a facility offering MAUD (difference, 1.90 pp; 95% CI, 1.26-2.54 pp; *P* < .001), while the difference associated with driving deaths involving alcohol was not statistically significant (0.02 pp; 95% CI, −0.26-0.30 pp; *P* = .89). The percentage of uninsured residents was also not associated with a significant difference in MAUD availability (−0.43 pp; 95% CI, −0.90 to 0.04 pp; *P* = .07). A 1-pp increase in the poverty rate was associated with reduced MAUD presence within a county (−0.66 pp; 95% CI, −0.93 to −0.38 pp; *P* < .001). A higher share of adults with a bachelor’s degree or higher was associated with increased presence of MAUD within a county (1.28 pp; 95% CI, 1.13-1.43 pp; *P* < .001). Additionally, counties with a larger population aged 65 years or older had significantly lower MAUD presence (−2.33 pp; 95% CI, −3.02 to −1.65 pp; *P* < .001), as did counties with a higher proportion of non-Hispanic White residents (−0.58 pp; 95% CI, −0.71 to −0.46 pp; *P* < .001). All statistically significant associations remained significant after Bonferroni adjustments. We found that county-level presence of facilities offering MAUD was not associated with alcohol-induced driving death rates but was associated with binge drinking rates. These associations were broadly consistent when we assessed associations between the number of SUDTFs offering MAUD conditional on a county’s having any SUDTFs with MAUD (eTable 1 in Supplement 1) and when we allowed for cross-county variation across states by removing state fixed effects (eTable 2 in Supplement 1).

**Table 2.** Marginal Effect Estimates From Logistic Regressions Characterizing Counties With Any SUDTFs Offering MAUD

Characteristic	Marginal effect (95% CI), pp^a^	*P* value^b^	Bonferroni-adjusted *P* value^b^	Pseudo-*R*^2^
Rurality (reference: metropolitan)				
Rural adjacent	−22.40 (−24.43 to −20.38)	<.001	<.001	.28
Rural remote	−23.64 (−25.72 to −21.56)	<.001	<.001	
Percentage of driving deaths involving alcohol, per 1-pp increase	0.02 (−0.26 to 0.30)	.89	>.99	.16
Prevalence of binge drinking, per 1-pp increase	1.90 (1.26 to 2.54)	<.001	<.001	.17
Percentage uninsured, per 1-pp increase	−0.43 (−0.90 to 0.04)	.07	.67	.16
Percentage below poverty level, per 1-pp increase	−0.66 (−0.93 to −0.38)	<.001	<.001	.16
Percentage with bachelor’s degree or higher, per 1-pp increase	1.28 (1.13 to 1.43)	<.001	<.001	.33
Percentage aged ≥65 y, per 1-pp increase	−2.33 (−3.02 to −1.65)	<.001	<.001	.24
Percentage non-Hispanic White, per 1-pp increase^c^	−0.58 (−0.71 to −0.46)	<.001	<.001	.23

Abbreviations: MAUD, medications for alcohol use disorder; pp, percentage points; SUDTF, substance use disorder treatment facility.

^a^ Data are at the county-year level. All models were weighted by population counts in a county-year and include state and year fixed effects with SEs clustered at the state level. Separate univariate logistic regressions were estimated for each covariate.

^b^ *P* values were multiplied by 9 and capped at greater than .99 for Bonferroni adjustments.

^c^ Included race and ethnicity categories were Hispanic, non-Hispanic Black, and other (included non-Hispanic American Indian or Alaska Native, non-Hispanic Asian, non-Hispanic Native Hawaiian or Other Pacific Islander, multiracial, and some other race).

## Discussion

To our knowledge, this study provides the first longitudinal assessment of MAUD presence at the US county level between 2017 and 2023 and associations between county characteristics and MAUD availability in SUDTFs through 2023. While the percentage of counties with any SUDTFs offering MAUD increased from 2017 to 2023, the growth rate leveled off after 2021. This plateauing may reflect several factors identified in qualitative studies. These include pandemic-era workforce shortages and burnout^29^ as well as persistent structural barriers in rural communities that constrain short-term expansion—such as fewer qualified prescribers, limited care coordination and referral networks, weaker pharmacy infrastructure, and greater travel distances.^30,31^ Given that prior research has found that the presence of SUDTFs in a county may lead to decreases in alcohol poisoning mortality, expansions of facilities offering MAUD may be warranted.^32^

A notable finding is that counties without any SUDTFs offering MAUD tended to be low-resource, rural counties. These counties had a higher share of at-risk populations with lower socioeconomic status, as measured by higher rates of uninsurance, higher poverty, older population, and lower educational attainment. This association is consistent with prior research that found that organizations might not prioritize the offering of MAUD due to perceived patient susceptibility to care interruptions.^33^ We also found that while the presence of SUDTFs offering MAUD was positively associated with binge drinking, MAUD availability was not associated with alcohol-induced driving deaths.

Compared with prior literature, our findings were generally consistent in identifying disparities in MAUD availability by socioeconomic and geographic factors. Using data from the National Directory of Drug and Alcohol Abuse Treatment Facilities from 2016 to 2019, 1 study found that facilities offering MAUD were more likely to be available in urban counties but not in counties with a higher prevalence of excessive drinking.^19^ Another study examined county-level factors associated with facilities offering MAUD in Medicare Part D for the years 2014 to 2018 and found that higher poverty, alcohol use disorder rates, and percentage of the population that was non-Hispanic White were associated with the availability of MAUD in Medicare Part D, but the study failed to detect a statistically significant association with rurality.^34^ Our study thus adds to a small but growing body of literature characterizing the evolving landscape of geographic access to SUDTFs offering MAUD by observing the most recent data available.^13,19,34^

### Limitations

This study has several limitations. First, the data were self-reported and subject to potential desirability or recall bias. Second, our measure of MAUD presence in a county did not capture facility capacity relative to use. Third, our data were based on voluntary listings of the National Directory of Drug and Alcohol Abuse Treatment Facilities, and not all facilities agree to be listed in the directory. As a result, our results are limited to facilities that agreed to be listed and are not generalizable to facilities that did not agree to be listed. However, over 90% of licensed SUDTFs are expected to be included in the directories.^35^ Fourth, this study was focused on associations between the presence of SUDTFs offering MAUD and county-level characteristics. None of the results should be interpreted as exhibiting causal relationships. Fifth, because the data were aggregated at the county level, associations between county characteristics and MAUD availability may differ from associations observed among individuals within counties, and thus they may be subject to an ecological bias.^36^ Sixth, medications not approved by the FDA for AUD are not included in MATTR. Future research can explore whether these barriers to access persist for MAUD not approved by the FDA.

## Conclusions

In this cross-sectional study of SUDTFs in US counties, the proportion of counties with at least 1 SUDTF offering MAUD increased from approximately 34% in 2017 to 44% in 2021 but plateaued thereafter. A continuation of efforts to expand the availability of facilities offering MAUD may be warranted. Policymakers may be interested in focusing their efforts to expand the presence of SUDTFs offering MAUD in rural counties with lower overall socioeconomic status.

## Notes

Supplement 1. **eFigure.** Number of SUDTFs Offering MAUD per 100 000 Population From 2017 to 2023 **eTable 1.** Negative Binomial Regression Estimates of the Association Between County Characteristics and the Number of SUDTFs Offering MAUD in a County, Conditional on at Least 1 SUDTF Present **eTable 2.** Marginal Effect Estimates From Univariate Logistic Regressions Examining the Association Between County Characteristics and the Probability That a County Has Any SUDTFs Offering MAUD, Without State Fixed Effects

Supplement 2.
Data Sharing Statement
