# Errors in Figures and Results

**DOI:** 10.1001/jamanetworkopen.2026.1178

**Published:** 2026-02-23

The Original Investigation titled “Medication Availability for Alcohol Use Disorder in Substance Use Disorder Treatment Facilities,”^1^ published on January 12, 2026, was corrected to fix errors in the figure keys for Figures 1 and 2. In Figure 1, the color representing the SUDTFs per county offering MAUD was transposed with the color representing SUDTFs offering MAUD. In Figure 2, the color representing No was transposed with the color representing Yes. In addition, in the Results there were errors in the percentages of patients offered acamprosate and disulfiram as well as errors in *P* values for the difference in driving deaths involving alcohol and for the percentage of non-Hispanic White residents. This article was corrected online.^1^
