# Supplementary material for: Medication Availability for Alcohol Use Disorder in Substance Use Disorder Treatment Facilities
Source: JAMA Netw Open. 2026 Jan 12;9(1):e2551563. doi: 10.1001/jamanetworkopen.2025.51563 (PMC12797095; doi:10.1001/jamanetworkopen.2025.51563)
Supplement: Supplement 1. — eFigure. Number of SUDTFs Offering MAUD per 100 000 Population From 2017 to 2023 eTable 1. Negative Binomial Regression Estimates of the Association Between County Characteristics and the Number of SUDTFs Offering MAUD in a County, Conditional on at Least 1 SUDTF Present eTable 2. Marginal Effect Estimates From Univariate Logistic Regressions Examining the Association Between County Characteristics and the Probability That a County Has Any SUDTFs Offering MAUD, Without State Fixed Effects [file jamanetwopen-e2551563-s001.pdf]

# Supplementary Online Content

Mizushima Y, Cantor J, McBain RK, et al. Medication availability for alcohol use disorder in substance use disorder treatment facilities. *JAMA Netw Open*. 2026;9(1):e2551563. doi:10.1001/jamanetworkopen.2025.51563

**eFigure.** Number of SUDTFs Offering MAUD per 100 000 Population From 2017 to 2023

**eTable 1.** Negative Binomial Regression Estimates of the Association Between County Characteristics and the Number of SUDTFs Offering MAUD in a County, Conditional on at Least 1 SUDTF Present

**eTable 2.** Marginal Effect Estimates From Univariate Logistic Regressions Examining the Association Between County Characteristics and the Probability That a County Has Any SUDTFs Offering MAUD, Without State Fixed Effects

This supplementary material has been provided by the authors to give readers additional information about their work.

© 2026 Mizushima Y et al. *JAMA Network Open*.

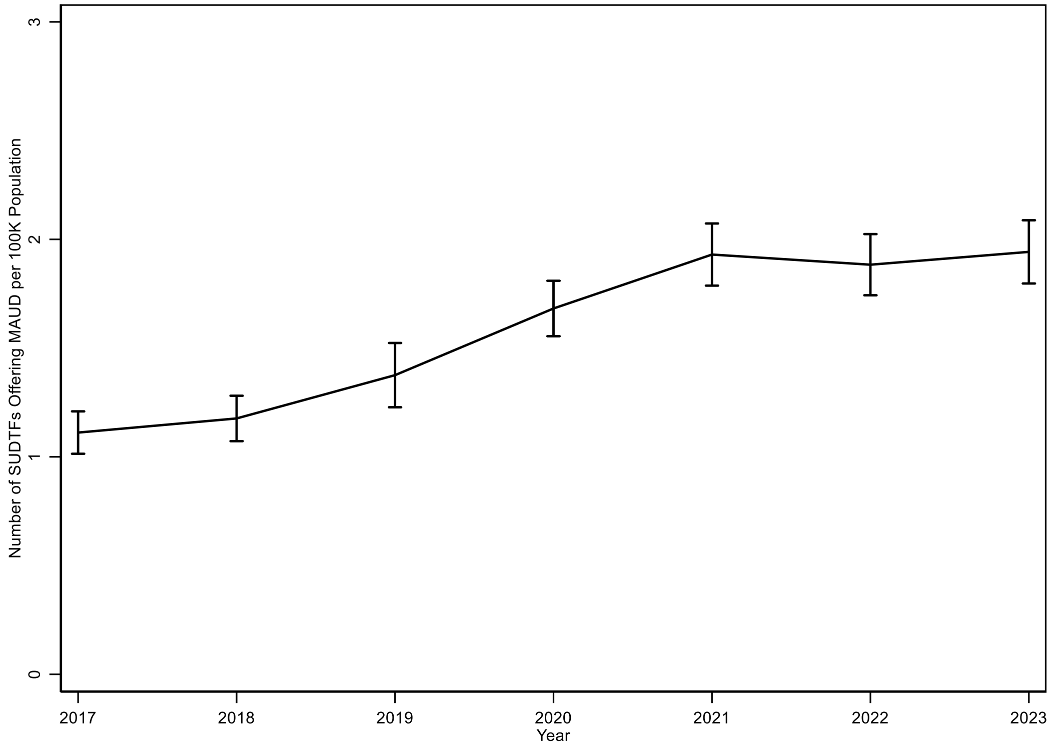


**eFigure Number of SUDTFs Offering MAUD per 100 000 Population From 2017 to 2023**

*Notes: The figure depicts the number of SUDTFs offering MAUD per 100,000 population in a county. T-tests comparing the coefficients for 2021 vs. 2022 and 2021 vs. 2023 were not statistically significant (P=.65 and P=.91, respectively).*

© 2026 Mizushima Y et al. *JAMA Network Open*.

eTable 1. Negative Binomial Regression Estimates of the Association Between County Characteristics and the Number of SUDTFs Offering MAUD in a County, Conditional on at Least 1 SUDTF Present

| | Estimate | 95% CI | P-Value | Bonferroni Adjusted P-Value | Pseudo R-Squared |
|---|---|---|---|---|---|
| Rural Adjacent (Reference: Metropolitan) | -162.45 | [-181.70,-143.19] | <.001 | <.001 | 0.10 |
| Rural Remote (Reference: Metropolitan) | -148.47 | [-171.62,-125.32] | <.001 | <.001 | |
| % Driving Deaths involving Alcohol | -3.17 | [-6.18, -0.16] | .04 | .35 | 0.09 |
| % Binge Drinking | 1.4 | [-4.29, 7.09] | .63 | 1 | 0.09 |
| % Uninsured | 12.01 | [2.92, 21.11] | .01 | .09 | 0.09 |
| % Below Poverty Level | 2.89 | [0.53, 5.25] | .02 | .15 | 0.09 |
| % with BA+ | 3.64 | [2.33, 4.95] | <.001 | <.001 | 0.10 |
| % 65 and Older | -11.22 | [-16.63, -5.82] | <.001 | <.001 | 0.10 |
| % Non-Hispanic White | -3.48 | [-4.61, -2.35] | <.001 | <.001 | 0.12 |

Notes*: Data are at the county-year level. Percentage points reported for all covariates. "Metropolitan" are counties with 2023 Rural-Urban Continuum Codes 1-3, "Rural Adjacent" are codes 4, 6, and 8, and "Rural Remote" are codes 5, 7, and 9. Each row is a separate univariate regression with state and year fixed effects. Connecticut uses 2023 RUCC codes for 2022-2023, and 2013 RUCC classification codes for 2017-2021. Estimates are weighted by population counts in a county-year. All models cluster standard errors at the state level.*

© 2026 Mizushima Y et al. *JAMA Network Open*.

eTable 2. Marginal Effect Estimates From Univariate Logistic Regressions Examining the Association Between County Characteristics and the Probability That a County Has Any SUDTFs Offering MAUD, Without State Fixed Effects

| | **Marginal Effect** | **95% CI** | **P-Value** | **Bonferroni Adjusted P-Value** | **Pseudo R-Squared** |
|---|---|---|---|---|---|
| Rural Adjacent (Reference: Metropolitan) | -25.38 | [-29.68,-21.08] | <.001 | <.001 | 0.17 |
| Rural Remote (Reference: Metropolitan) | -27.97 | [-31.57,-24.38] | <.001 | <.001 | |
| % Driving Deaths involving Alcohol | -0.09 | [-0.46,0.28] | .63 | 1 | 0.01 |
| % Binge Drinking | 1.93 | [0.83, 3.03] | .001 | .004 | 0.03 |
| % Uninsured | -0.84 | [-1.39, -0.30] | .002 | .03 | 0.02 |
| % Below Poverty Level | -0.99 | [-1.39, -0.58] | <.001 | <.001 | 0.03 |
| % with BA+ | 1.45 | [1.23, 1.67] | <.001 | <.001 | 0.22 |
| % 65 and Older | -1.57 | [-2.70, -0.44] | .007 | .03 | 0.05 |
| % Non-Hispanic White | -0.41 | [-0.53, -0.30] | <.001 | <.001 | 0.07 |

Notes*: Data are at the county-year level. Percentage points reported for all covariates. "Metropolitan" are counties with 2023 Rural-Urban Continuum Codes 1-3, "Rural Adjacent" are codes 4, 6, and 8, and "Rural Remote" are codes 5, 7, and 9. Connecticut uses 2023 RUCC codes for 2022-2023, and 2013 RUCC classification codes for 2017-2021. Race categories included Black alone, non-Hispanic; Hispanic; White alone, non-Hispanic; and Other. "Other" includes American Indian and Alaska Native, non-Hispanic; Asian, non-Hispanic; Native Hawaiian and Other Pacific Islander, non-Hispanic; "Some other race", non-Hispanic; and two or more races. P-values were multiplied by 9 and capped a 1 for Bonferroni adjustments. Each row is a separate univariate regression with state and year fixed effects. Estimates are weighted by population counts in a county-year. All models cluster standard errors at the state level.*

© 2026 Mizushima Y et al. *JAMA Network Open*.
