# Supplementary material for: Medication Availability for Alcohol Use Disorder in Substance Use Disorder Treatment Facilities
Source: JAMA Netw Open. 2026 Jan 12;9(1):e2551563. doi: 10.1001/jamanetworkopen.2025.51563 (PMC12797095; doi:10.1001/jamanetworkopen.2025.51563)
Supplement: Supplement 2. — Data Sharing Statement [file jamanetwopen-e2551563-s002.pdf]

Data Sharing Statement

Mizushima. Medication Availability for Alcohol Use Disorder in Substance Use Disorder Treatment Facilities. *JAMA Netw Open*. Published January 12, 2026.
doi:10.1001/jamanetworkopen.2025.51563

Data
Data available: Yes
Data types: Deidentified participant data, Data (not involving human participants)
How to access data: ymizushi@rand.org
When available: With publication

Supporting Documents
Document types: Statistical/analytic code
How to access documents: ymizushi@rand.org
When available: With publication

Additional Information
Who can access the data: researchers whose proposed used of the data has been approved
Types of analyses: reproduction purposes
Mechanisms of data availability: with investigator support
